# Homologous Recombination as a Fundamental Genome Surveillance Mechanism during DNA Replication

**DOI:** 10.3390/genes12121960

**Published:** 2021-12-09

**Authors:** Julian Spies, Hana Polasek-Sedlackova, Jiri Lukas, Kumar Somyajit

**Affiliations:** 1 Novo Nordisk Foundation Center for Protein Research, Protein Signaling Program, Faculty of Health and Medical Sciences, University of Copenhagen, Blegdamsvej 3b, DK-2200 Copenhagen, Denmark; julian.spies@cpr.ku.dk (J.S.); hana.sedlackova@cpr.ku.dk (H.P.-S.); 2 Functional Genomics and Metabolism Research Unit, Department of Biochemistry and Molecular Biology, University of Southern Denmark, Campusvej 55, DK-5230 Odense, Denmark

**Keywords:** DNA replication, replication stress, homologous recombination, RAD51, BRCA1/2, replication fork protection, daughter strand gaps, DNA polymerases, chromosome stability, cancer microenvironment, adaptative mutagenesis, cancer evolution, cancer therapy

## Abstract

Accurate and complete genome replication is a fundamental cellular process for the proper transfer of genetic material to cell progenies, normal cell growth, and genome stability. However, a plethora of extrinsic and intrinsic factors challenge individual DNA replication forks and cause replication stress (RS), a hallmark of cancer. When challenged by RS, cells deploy an extensive range of mechanisms to safeguard replicating genomes and limit the burden of DNA damage. Prominent among those is homologous recombination (HR). Although fundamental to cell division, evidence suggests that cancer cells exploit and manipulate these RS responses to fuel their evolution and gain resistance to therapeutic interventions. In this review, we focused on recent insights into HR-mediated protection of stress-induced DNA replication intermediates, particularly the repair and protection of daughter strand gaps (DSGs) that arise from discontinuous replication across a damaged DNA template. Besides mechanistic underpinnings of this process, which markedly differ depending on the extent and duration of RS, we highlight the pathophysiological scenarios where DSG repair is naturally silenced. Finally, we discuss how such pathophysiological events fuel rampant mutagenesis, promoting cancer evolution, but also manifest in adaptative responses that can be targeted for cancer therapy.

## 1. Introduction

Accurate and timely propagation of genetic information across multiple generations of dividing cells is a fundamental process initiated by DNA replication and completed by segregation of intact and complete copies of the genome to the progeny [1,2]. The process of DNA replication relies on precise coordination of numerous enzymatic activities congregated within the replisome, a molecular machine in charge of unwinding the parental DNA template and synthesizing nascent DNA strands [2]. Given the complexity of the cellular milieu and varying growth conditions of different cell types, the flawless movement of the replisome is constantly challenged by various genotoxic stresses of exogenous and endogenous origins, including metabolic stress accompanying perturbed nucleotide levels, lesions on template DNA, secondary DNA structures, or transcription complexes encountered by a moving replisome [3,4,5]. Replication perturbations that escape genome surveillance mechanisms and continue to cause pausing or stalling of progressing replisomes cause replication stress (RS), a key driver of genomic instability with causal links to developmental disorders, accelerated aging, tissue degeneration, and tumorigenesis [5,6,7]. To counteract endogenous and exogenous sources of RS, including those caused by commonly used chemotherapeutic regimens, cells mount replisome-based genome surveillance that orchestrates cell cycle progression, replication initiation, replication fork stability, and DNA repair [5]. Due to its central importance for genome stability, replication-coupled genome surveillance entails remarkable replisome plasticity, such as DNA replication speed modulation, engaging specialized enzymatic activities that remodel and mediate the stability and restart of stalled replication forks and triggering ATR-mediated cell cycle checkpoint response [6]. The plasticity of replisomes spanning acute responses to various forms of sub-lethal replication perturbations and the role of replication checkpoints have been recently covered in excellent review articles [5,6,8,9,10,11]. In this manuscript, we set out to complement these by discussing the unifying role of replication-coupled homologous recombination (HR) as a key DNA repair mechanism that constantly surveys replicating genomes in human somatic cells. Specifically, we focused on the DNA double-strand break (DSB)-independent role of the recombinase RAD51 and RAD51 mediators, enabling the protection and restart of stalled replication forks and continuous DNA synthesis across DNA lesions bypassed by the replisome. We examined the recently reawakened interest in ssDNA gaps behind the fork (also known as daughter strand gaps, DSGs) as a major form of genomic lesions arising during unperturbed DNA replication and upon non-stalling replication interferences. We highlighted various cell-intrinsic and -extrinsic sources of DSGs in human somatic cells and reviewed the spatial and mechanistic aspects of HR-mediated repair of DSGs in the vicinity of an active replisome. Finally, we discussed the cellular context and the pathological outcome of impaired HR-mediated DSG repair in fueling cancer adaptive responses during early stages of cancer evolution and as a response to cancer therapy.

## 2. Homologous Recombination: An Overview

HR is an evolutionary, conserved, fundamental DNA repair process that plays a central role in persevering genome integrity and completion of DNA replication [12,13,14,15]. Conversely, dysfunction of HR is a prime cause of chromosomal instability in mammalian somatic cells characterized by an under-replicated genome, increased genomic mutational burden, and aberrant genetic rearrangements (such as chromosomal translocations, deletions, amplifications, or loss of heterozygosity, LOH) [16]. The intricate links between HR-mediated repair, genomic instability, and human diseases marked by unstable genomes, particularly cancer, have been widely appreciated ever since the identification of two major hereditary breast/ovarian cancer predisposition genes, *BRCA1* and *BRCA2* [17,18]. A thorough genetic, biochemical, and cell-based characterization of BRCA1 and BRCA2 established them as central regulators of RAD51, a central recombinase involved in HR, thereby linking HR with tumor suppression [19,20,21,22,23,24,25]. Shortly after these seminal discoveries, other cancer types and cancer susceptibility genetic diseases, such as Ataxia Telangiectasia, Nijmegen breakage syndrome, Bloom syndrome, and Fanconi anemia (FA), showed a strong correlation to the germline mutations in several other HR proteins engaged in HR or functional mutations of single genes that result in hyper- or hypo-recombination phenotypes [26,27]. Collectively, these findings established a crucial role of HR and its regulators in tumor suppression.

Although the role of HR is extensively characterized for the repair of DSBs, intensive research during the last decade has also identified HR as a pivotal caretaker of DNA replication under stress [14,28]. Indeed, multiple HR factors were shown to play central roles in protecting nascent DNA strands, promoting the restart of stalled replication forks, preventing the collapse of replication forks, orchestrating repair of DNA interstrand cross-links (ICLs) in collaboration with nucleotide excision repair or translesion synthesis (TLS), and surveying DSGs for their repair during stochastic replication errors [6]. The unifying knowledge arising from insights into HR-mediated DSB repair and DNA replication has been extremely rewarding, not least by paving ways to better understand how defects in HR confer tumor susceptibility and exploiting defective HR to enhance various forms of chemotherapy, including inhibitors of poly (ADP-ribose) polymerase (PARP) [29]. Because of its central bearing in this review, we start with a mechanistic overview of all the key steps involved in HR.

### Critical Determinants and Mechanistic Basis of HR in a Nutshell

**Figure 1:**
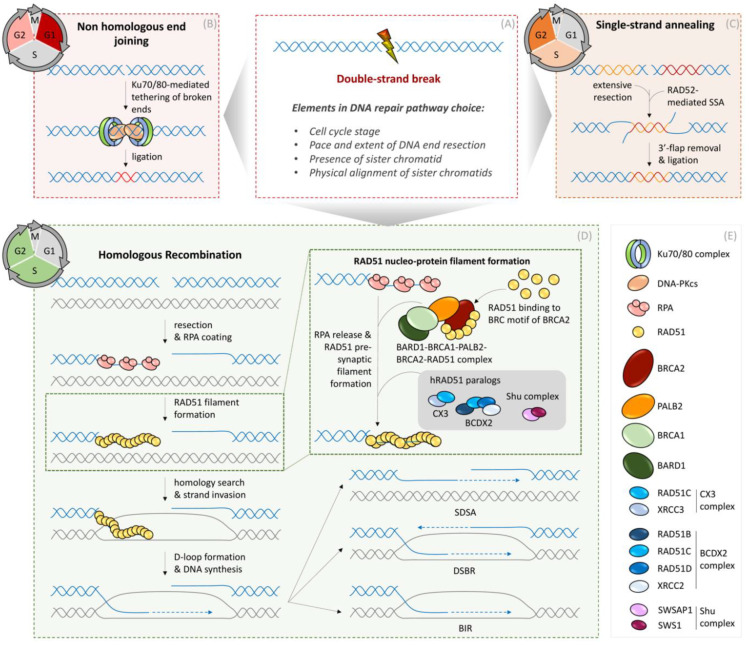
Mechanisms of DNA double-strand break repair in somatic cells. (**A**) DNA double-strand break repair pathway choice is driven by many factors such as cell cycle stage, availability of sister chromatids, and other chromatin-shaping elements. (**B**) Non-homologous end-joining (NHEJ) is the predominant DSB repair pathway in G1 phase, but also operates throughout the remaining interphase of the cell cycle. Ligation of DSB ends is mediated by the central NHEJ factors Ku70/80 and DNA-PKcs. Prior to ligation, processing of DSB ends and regulation of the ligation reaction is orchestrated by additional DNA repair factors. (**C**) Single-strand annealing (SSA) operates in S and G2 phases of the cell cycle when extensive resection at the DSB ends can be employed for generating ssDNA patches. Annealing of ssDNA is catalyzed by the RAD52 recombinase and down-stream processing of 3′-flaps and ligation of DNA is mediated by additional specialized DNA repair/replication factors. (**D**) Homologous Recombination (HR) is limited by the availability of an intact sister chromatid serving as a template to restore the lost genetic information. Therefore, HR operates in S and G2 phases of the cell cycle after DNA replication generates homologous sister chromatids. Initially, DSB ends are resected, and the resulting 3′ ssDNA overhangs are bound by Replication Protein A (RPA). RPA is subsequently replaced by the RAD51 recombinase, leading to the formation of a RAD51 nucleo-protein filament. RAD51 recruitment to ssDNA is mediated by BARD1, BRCA1, PALB2, and BRCA2 proteins and stabilization of RAD51 filaments is promoted by BRDX2, CX3, and Shu complexes (see protein composition of complexes in box (**E**)). Stable RAD51 filaments search for homologous sequences in the sister chromatid, and subsequent strand invasion into the homologous sister chromatid and D-loop formation are fundamental steps before enabling DNA repair synthesis. After DNA synthesis, two sub-pathways of HR can be employed. During Double-strand break repair (DSBR), DNA repair synthesis promotes D-loop extension and second end capture, resulting in double Holiday Junctions (dHJ), which are either dissolved or resolved by specialized enzymes, resulting in either non-crossing-over or crossing-over products, respectively. In the case of the second HR sub-pathway, called synthesis-dependent strand annealing (SDSA), nascent DNA is displaced from the D-loop and anneals with the second end of the DSB, resulting in non-crossing-over products. In the case of a broken replication fork, if a second DSB end is not available, break-induced replication (BIR) can occur, using the intact sister chromatid as a template. (**E**) Legend of symbols for respective DNA repair enzymes.

Together with non-homologous end joining (NHEJ), HR is one of the two major pathways for the timely repair of DSBs in mammalian cells [30]. Unlike NHEJ, which involves a rapid and efficient ligation of DNA ends with minimal processing of DNA ends (Figure 1), HR is subjected to additional layers of highly regulated steps. The major global determinant of HR in somatic cells is the cell cycle. HR is primarily restricted to the S and G2 phase of cycling cells and utilizes an intact homologous sequence of the duplicated sister chromatid as a template for a precise repair reaction. Extensively studied in the context of DSB repair, the mechanistic basis of HR involves the following three steps.

First, the processing of DNA ends is initiated by the MRN complex comprising MRE11, RAD50, and NBS1 (ortholog of NBS1 in *Saccharomyces cerevisiae* is XRS2) together with CtIP (Sae2 in *Saccharomyces cerevisiae*) [31]. The initial DNA-end resection is then further fueled to generate an extensive 3′ ssDNA tail by the 5′-3′ exonuclease Exo1 in concert with the helicase/nuclease activities of BLM/DNA2. Multiple layers of DNA damage-signaling regulators are integrated into this initial stage of HR to optimize the speed and extent of DNA end processing in order to generate DNA substrates that can be utilized in the subsequent strand exchange reactions. As such, DNA-end processing is an extremely critical determinant of DNA repair pathway choice, and its extent inversely correlates with DNA repair fidelity [30,31,32]. For instance, partially processed DNA ends favor alternative end-joining (also known as microhomology end-joining), which is substantially more mutagenic than classical NHEJ. Conversely, excessive DNA-end resection can result in a switch between high-fidelity HR to single-strand annealing (SSA), which involves RAD52 recombinase-dependent deleterious annealing of complementary strands and is, thus, highly mutagenic [32,33] (Figure 1).

The 3′ ssDNA resulting from DNA end processing is occupied by an abundant replication protein A (RPA complex, including RPA1, RPA2, and RPA3). This step is crucial for functional HR as RPA-bound ssDNA facilitates the opening of secondary structures in ssDNA for efficient HR, as well as preventing spurious pairing of resected ssDNA ends [31]. Moreover, being a central activator of checkpoint signaling, RPA bound on resected DNA also serves as a recruitment platform for several checkpoint proteins and their regulators [5]. However, RPA bound on ssDNA also poses a barrier to RAD51 loading, and, thus, RPA must be replaced with RAD51 before HR proceeds to the next steps.

Second, the exchange of RPA with RAD51 recombinase leads to the formation of presynaptic nucleoprotein filament, followed by homology search and strand [27,34]. The vertebrate RAD51 belongs to a RAD (radiation repair genes) superfamily of recombinases RADα (RAD51, DMC1) and RADβ (RAD51 paralogs including RAD51B, RAD51C, RAD51D, XRCC2, and XRCC3) [34]. RAD51 is present across all kingdoms of life and shares common origin of the bacterial recA superfamily [34]. Its recruitment and nucleation on RPA-coated ssDNA is a rate-limiting step, which requires the aid of the RAD51 mediators to facilitate RPA displacement from resected ssDNA ends. This is achieved through dedicated mediators comprising BRCA2 (functionally analogous to Rad52 in *Saccharomyces cerevisiae*) with the 26S proteasome complex subunit DSS1 and the BRCA1–BARD1 complex bound to the partner and localizer of BRCA2 (PALB2) [27]. In a highly coordinated fashion, these mediators bind both ssDNA and RAD51 monomers and replace RAD51 with RPA on the resected ssDNA (Figure 1). Interestingly, BRCA1 is involved both in the DNA end resection step and RAD51 loading, posing the need for a highly coordinating coupling between these two early stages of HR [30].

Other essential RAD51 mediators include the RAD51 paralogs (RAD51B, RAD51C, RAD51D, XRCC2, and XRCC3). RAD51 paralogs were initially identified by a genome-wide database search as proteins with 20–30% sequence identity with human RAD51, mainly restricted to ATP-binding and hydrolysis Walker A and Walker B motifs, respectively [34,35]. Since then, a large body of genetic and biochemical studies revealed that mammalian RAD51 paralogs form two functionally distinct complexes: the BCDX2 complex (with RAD51B-RAD51C-RAD51D-XRCC2) and the CX3 complex (with RAD51C- XRCC3), which facilitate loading and stabilization of RAD51 onto the ssDNA, remodeling the RAD51 nucleoprotein complex during stand invasion and homology search, thereby fostering completion of HR [36,37,38,39,40,41,42] (Figure 1). Besides augmenting the stable RAD51-nucleoprotein filament, human RAD51 paralogs were recently implicated in promoting BRCA2-independent meta-stable RAD51 filaments, capable of promoting replication fork reversal during RS [43]. In addition to the RAD51 paralogs, the human Shu complex and the recently identified SWSAP1 protein, which shares amino acid sequence identity with the archaeal recombinase RadA, were also shown to mediate RAD51 during the early stages of HR [34] (Figure 1).

Once formed, the RAD51–ssDNA nucleoprotein filaments become dynamic structures that undergo a quality control mechanism through an equilibrium of their assembly and disassembly rates. Indeed, several motor proteins, including RECQL5, FBH1, Fanconi anemia group M protein (FANCM), and regulator of telomere elongation helicase 1 (RTEL1), oppose RAD51 mediators at various stages and promote RAD51 filament disassembly, thus providing a quality control against aberrant, untimely, or excessive usage of recombination reactions [30,44].

Third, the presynaptic filament of RAD51–ssDNA nucleoprotein engages in homology search, invades homologous DNA duplex, and initiates base-pairing with the complementary sequence at the sister chromatid. The BRCA1–BARD1 complex and RAD51 paralogs facilitate homologous pairing and formation of synaptic complexes with a three-stranded DNA helix intermediate composed of the invading strand and the complementary strand of the invaded molecule [27]. Once base-pairing is established, the non-base-paired strand of the invaded molecule is displaced to form a D-loop (Figure 1). This step is driven by RAD51-mediated ATP hydrolysis and accompanies RAD51 filament disassembly and initiation of DNA synthesis at the free 3′ end of the invading strand. The motor protein RAD54 facilitates the unloading of RAD51 during D-loop formation and late HR structures to promote DNA repair synthesis. The D-loop engages replicative or TLS DNA polymerases, which extend the invading strand using the donor DNA molecule from sister chromatid as a template in a process known as gene conversion. The recombination intermediate can be channeled into two sub-pathways at this D-loop extension step: DNA double-strand break repair (DSBR) or synthesis-dependent strand annealing (SDSA) [45,46] (Figure 1). In the DSBR sub-pathway, the extended D-loop can be captured by the second end of the DSB, which results in a double Holliday junction (HJ). A junction-specific endonuclease can then resolve the double HJs to generate crossover or non-crossover products. Although crossover-associated recombination is essential for proper chromosome disjunction in meiotic cells, this pathway is suppressed in somatic cells to avoid crossover-associated LOH and chromosome rearrangements [45]. The concerted action of helicases and topoisomerases (Bloom Syndrome helicase, BLM together with the type IA topoisomerase Topo IIIα) are implicated in dissolving double HJs into non-crossover recombination products [45]. In the SDSA sub-pathway, which is the predominant pathway in somatic cells, the nascent DNA strand is displaced from the D-loop and subsequently anneals with the second end of the DSB to resolve as a non-crossover product [47] (Figure 1).

## 3. HR as an Intrinsic Safeguard of DNA Replication in Mammalian Somatic Cells

Besides the functions of HR during DSB repair, its role during DNA replication, both normal and especially under various forms of stress, has gained increasing attention. Among the key conclusions of this nearly decade-long research is the notion that during physiological cell growth, the most prominent role of RAD51 and its upstream regulators is safeguarding the stability of replication forks [6]. In the subsequent sections, we will focus on the crucial but hitherto largely neglected quantitative features of RS. We will categorize RS based on its severity as low, moderate, or severe, and systematically dissect how HR, in general, and RAD51, in particular, mitigate deleterious consequences associated with increasing impediments of DNA replication. At the end of the review, we will synthesize the most recent findings in this area in a conceptual framework including their implication in cancer development.

### 3.1. Low RS Coupled to Replication Fork Slowing: RAD51 and Beyond

**Figure 2:**
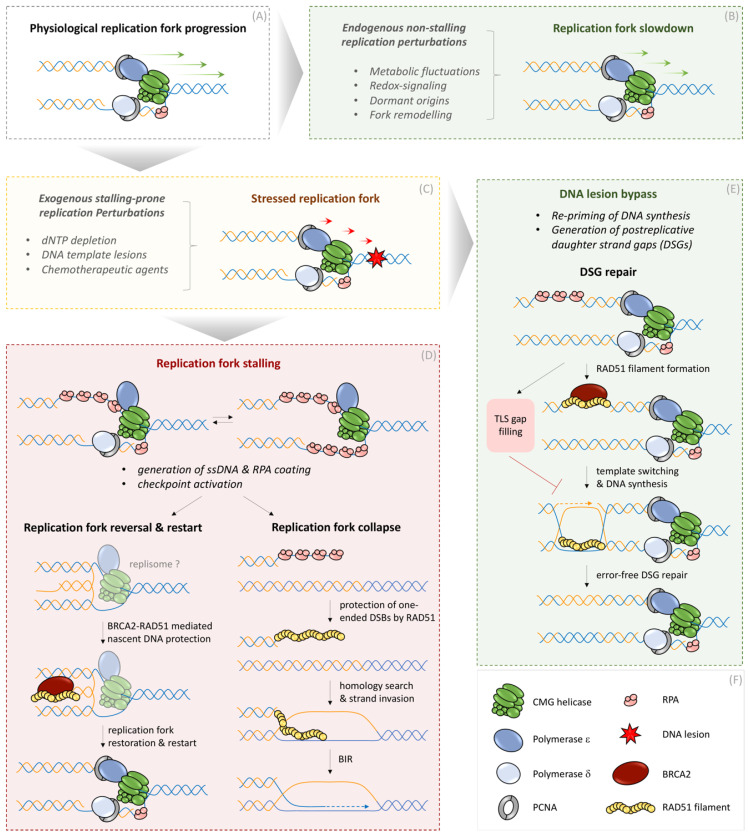
Replisome-based response to replication perturbations. (**A**) Unperturbed replication without endogenous or exogenous stresses is defined by physiological fork progression rates, combining high fidelity with ‘fast’ DNA replication. (**B**) Low replication stress: Endogenous sources of stress marked by metabolic fluctuations, redox signaling, dormant origins, or remodeled replication forks can slow down replication fork speed. (**C**) High replication stress: Exogenous sources of replication stress are represented by dNTP depletion, DNA template lesions, and chemotherapeutic agents. Depending on the nature of replication perturbation, stress at the replication fork can be resolved by DNA lesion bypass or lead to replication fork stalling. (**D**) Upon replication fork stalling, RPA loaded on ssDNA activates checkpoint signaling. Replication fork reversal and restart: Stalled replication forks can be actively reversed into a four-way junction DNA structure, mediated by the catalytic activity of several translocases. Nascent ssDNA within such reversed fork structures is protected by BRCA2-RAD51 from further nucleolytic attacks. Upon stress release, reversed replication forks can be restarted by conversion into active three-way junction replication forks through a RAD51-dependent recombination mechanism. Replication fork collapse: Upon prolonged fork stalling, destabilized forks can collapse, resulting in one-ended DSB. Resection of the DNA break end is followed by RAD51 loading, promoting homology search and strand invasion further repaired by BIR, as outlined in Figure 1. (**E**) Moderate replication stress: Among the most common challenges for replication forks is the blockage of fork passage by, e.g., bulky adducts, caused by endogenous metabolites or by exogenous reactive compounds. Mammalian cells can bypass this type of DNA lesion by re-priming behind the DNA lesion. Re-priming can leave ssDNA gaps, named daughter strand gaps (DSGs), behind replication forks. Such DSGs are repaired following the basic steps of HR: RAD51 loading on ssDNA gaps, a template switching involving the intact sister chromatid as a template and, finally, error-free DSG repair synthesis. Moreover, RAD51-dependent DSG repair protects from translesion DNA synthesis (TLS)-mediated gap filling at sites of DSGs. (**F**) Legend of symbols for respective DNA repair enzymes.

As a first response mechanism to low levels of RS, such as physiological assaults originating from topological stress or metabolic fluctuations, active replication forks can sense such perturbations in a RAD51-dependent or -independent manner and react by the global slowdown of replication fork speed to counteract an aggravation of a more severe stress level [6] (Figure 2). During every cell cycle, replication forks face a variety of threats that challenge faithful DNA duplication. These include, but are not limited to, metabolic imbalance inflicting nucleotide fluctuations, topological stress ahead of or behind the replisome, or unavailability of rate-limiting components required for physiological pace of DNA replication [5]. The replisome has the unique property to distinguish these low but constant forms of RS sources and respond to them simply by altering the replication fork speed (Figure 2). It is now well established that cells can tolerate modulation of fork speed within a defined dynamic range, which serves as key genome surveillance mechanism and guards against more severe forms of RS [6]. Such replisome speed plasticity is achieved via two major routes, active reorganization of the replisome motor and reversible remodeling the DNA structure underneath the replisome, respectively. For instance, mild attenuations of metabolic nucleotide biosynthesis that is associated with the generation of signaling-competent reactive oxygen species (ROS) is sensed by the replisome-associated ROS sensor PRDX2 and is relayed to adjustment of replication speed by transiently unrooting the replisome accelerator TIMELESS [48]. This, in turn, triggers an instant yet fully reversible fork slow-down and, thus, protection of the replicating genome against nucleotide fluctuations. Interestingly, besides accessory replisome components (PRDX2, TIMELESS), replisome speed modulations also involve dynamic alterations of core replisome components including CDC45 (checkpoint-dependent phosphorylation in response to RS) [49] or GINS (during DNA ICL repair) [50]. Furthermore, architectural changes of the replisome with direct impact on the speed of DNA replication are well known to entail posttranslational modifications (ubiquitylation, sumoylation) of PCNA, a processivity factor for replisome-associated DNA polymerases [6,51].

Besides replisome structure, studies pioneered by the Lopes laboratory have established that reversible remodeling of replication forks (often referred to as ‘fork reversal’), which was originally considered largely as a hallmark of extreme RS, can exercise genome protective replication also under low RS via multiple ways, inducing fork slowdown [52]. Specifically, it was shown that RAD51, in a RAD51 paralog-dependent but BRCA2-independent fashion, can remodel replication fork into a four-way chicken foot-like structure [43]. It has been proposed that RAD51-mediated fork reversal is a noncanonical recombination process that does not require stable RAD51-nucleoprotein filaments, but rather depends on meta-stable discontinuous RAD51 filaments [15], whose dynamics are highly regulated by RPA-like single-strand DNA-binding protein, X-linked (RADX), F-box helicase 1 (FBH1) or RecQ-like helicase 5 (RECQ5) [6,53]. Remodeling of replication forks reduces the rate of active fork progression, which is fully reversible and can be rapidly restored to normal fork speed in a RecQL1-dependent manner, which, in turn, is actively restrained by the polyADPribosylation activity of PARP1. In addition, several other annealing helicases and translocases such as SMARCAL1, ZRANB3, and HLTF are also associated with actively inducing fork reversal and restraining fork speed [6,54]. Whether these annealing helicases generate a DNA substrate, which is stabilized or remodeled by RAD51 recombinase or vice versa, is among the exciting open questions for future investigations. For instance, it will be interesting to know if the alterations in replisome architecture and RAD51-induced fork remodeling are mechanistically linked or if they operate in parallel ways by sensing RS of different nature or threshold. Furthermore, it is likely that replisome rearrangements associated with DNA topological stress are causing fork rotation [55], which might require RAD51-mediated fork reversal and replisome stabilization. Currently, it is also unknown how the integrity of the core replisome is maintained during RAD51-mediated fork reversal. Besides the catalytic power of RAD51 to remodel replication forks under low replication stress, RAD51 is also associated with the replisome (in particular, with the Polα complex) under unchallenged conditions [56]. In this regard, it is possible that the presence of RAD51 at transiently regressed forks might be required to stabilize replication-associated ssDNA/dsDNA junctions and thereby prevent uncontrolled uncoupling of the core replisome components.

Interestingly, ATR-mediated checkpoint signaling has also been implicated to inflict RAD51-mediated fork reversal and promote global fork slowdown in the presence of UV-induced DNA damage [57]. These results are intriguing as they challenge a well-established notion that reversal of replication forks represent a pathological RS scenario with irreversible fate of the affected replication forks as an upstream source of checkpoint signaling [8]. Rather, these new findings suggest that controlled fork reversal is genome protective and integrates global checkpoint signaling to a local fork dynamic via RAD51. In support of this hypothesis, RAD51 and its paralogs are shown to be phosphorylated by ATR in a fashion that stimulates their role in HR and DNA damage signaling [58,59,60]. Notably, other enzymes that promote fork reversal such as SMARCAL1 have been shown to be phosphorylated and restrained by ATR, reinforcing the notion that excessive fork reversal could be genotoxic and promote events such as replication catastrophe [61]. Finally, recent development in the RS field adds additional players for replication fork dynamics that might be integrated to the already intricate mechanisms described in this section. For instance, chromatin-bound Mini Chromosome Maintenance (MCMs) rings in the form of dormant origins [62], the number of active replication origins at any given time during S-phase [63], the coordination of DNA synthesis between leading and lagging strand, and the levels of p21 and PARP1 activity [64] may all contribute to the fine-tuning of the speed with which eukaryotic cells replicate their genomes. Whether and how these mechanisms cooperate with replisome architectural changes or RAD51-mediated remodeling of DNA structures at replication forks are currently unknown.

### 3.2. Severe RS and Global Stalling of Replication Forks: HR-Mediated Fork Stabilization and Replication Restart

In strong contrast to low RS, severe RS impedes active replication fork progression, resulting in complete fork stalling and eventually fork collapse (Figure 2). These harsh conditions of RS occur mainly under non-physiological treatments, e.g., during chemotherapy, and, therefore, we will only briefly portray RAD51 functions during such treatments.

In complete contrast to low RS, resulting in a slower but steady rate of DNA synthesis, high levels of RS are usually featured by complete replication stalling accompanied by exposure of ssDNA or dsDNA ends [5]. This makes stalled forks vulnerable to degradation by DNA nucleases such as MRE11, CtIP, DNA2, EXO1, and MUS81, causing DNA breakage and genome instability [28]. To safeguard genome integrity under such conditions, cells mobilize HR factors, including BRCA1, BRCA2, RAD51, RAD51 paralogs, and Fanconi anemia pathways’ proteins, which all synergize to protect highly vulnerable nascent DNA during persistent fork stalling [28,53,65,66,67,68,69,70] (Figure 2). If this level of protection fails, stalled replication forks upon extensive remodeling are subjected to pathological degradation of nascent DNA. As alluded to above, RAD51 is involved in both processes, but how it balances the two respective activities remains still unclear.

It is important to note that the mechanistic models of nascent DNA protection have been derived from studies based on acute treatment of cells with hydroxyurea (HU). HU inhibits ribonucleotide reductase (RNR), which, in addition to fork stalling, profoundly alters cellular metabolism by liberating signaling-competent ROS [48,71]. However, the impact of ROS signaling on nascent DNA integrity was long neglected. In a recent study, we showed that metabolic ROS during dNTP perturbation triggers ATM-mediated phosphorylation and activation of MRE11 and thereby instigates the degradation of stalled forks that lack their HR-mediated protection [69]. This finding highlights the importance of metabolic perturbations as an important contributor to nascent DNA instability in HR-deficient cells and implicates fork protection as a safeguard tool for (patho)physiological conditions marked by metabolic plasticity such as early development, oncogenic transformation, and cancer treatment. For instance, HR-deficient tumors are hypersensitive to various forms of chemotherapy, and restoration of fork protection is among the key sources of acquired resistance to such treatments [72,73,74]. Similarly, loss of key drivers of fork degradation such as PTIP or PARP1 rescues the lethality of BRCA2-deficient mouse embryonic stem cells, underscoring the critical role of fork protection in early development [73,74].

Besides fork remodeling and nascent DNA protection, HR, in general, and RAD51, in particular, have been thoroughly studied to promote recombination-dependent restart of forks upon chronic replication arrest (e.g., 24 h of HU treatment) [75,76]. Such a mechanism entails fork breakage into one-ended DSBs by structure-specific nucleases (MUS81), accompanied by RAD51 foci formation at sites of one-ended DSBs, thereby promoting repair and recovery of replication fork arrest [75,76] (Figure 2). Interestingly, in other biological scenarios, where oncogenes induce RS [77] or during the repair synthesis of under-replicated genome in mitosis [78], replication recovery is not dependent on RAD51 but rather on RAD52, facilitating an RNA-dependent repair mechanism. Since oncogene-induced replication stress also originates from transcription/replication conflicts [5,79], the presence of RNA at sites of fork breakage might explain the preference for RAD52-mediated break-induced replication [80].

Taken together, apart from responding to a low RS, HR can be mobilized to stabilize, repair, and restart stalled forks when harsh forms of RS are encountered. Paradoxically, cancer cells also utilize these mechanisms and confer resistance to chemotherapeutic drugs that induce high-level RS [70]; thereby, selective inhibition of replication fork protection mechanisms could be exploited as potential therapeutic strategies for targeting cancer cells, which are difficult to treat via conventional chemotherapies.

### 3.3. Moderate Replication Stress: HR-Mediated Genome Surveillance behind the Replication Fork

In contrast to severe RS, which is rare during physiological DNA replication and where all replication forks are globally stalled, ‘moderate forms’ of RS are frequent in somatic cells and usually tolerated and bypassed by a progressing replisome (Figure 2), and, thus, differ also from low RS responses, which are dealt with directly at the active replisome, as described above (Section 3.1). Because of these unique features of moderate RS and the fact that it has not yet been extensively reviewed, we discuss this important topic in more detail. Moderate RS can originate from disruptive DNA replication across bulky DNA adducts, inter- or intra-strand crosslinked DNA, or DNA torsional stress [6]. All these forms of RS avoid permanent replication block, thereby preventing fork instability or episodes of genome under-replication. On a mechanistic level, this involves bypassing of the DNA lesion, followed by repriming of the replisome [11] behind such a lesion (Figure 2). In consequence, such a mechanism generates nascent ssDNA gaps behind the replication fork (also known as daughter strand gaps, DSGs) [11], which are repaired by a specialized form of HR, the RAD51-mediated strand exchange [81]. Given the importance of DSG lesions in understanding replication stress during tumor formation, tumor evolution, and anti-cancer treatment, the sources of DSGs, their repair, and the consequences of unrepaired DSGs have recently gained tremendous attention [82,83].

#### 3.3.1. DSGs and Their Repair

Historically, the notion of DSG repair was established by the observations that *Escherichia coli* treated by ultraviolet (UV) light exhibited both the appearance of discontinuous DNA replication within newly replicated leading and lagging daughter strands and their repair behind replication fork passage [84,85]. These observations were consolidated in yeast and vertebrate models by combining various genetic, biochemical, and cell biology techniques including visual DSG capture by electron microscopy [57,86,87,88].

More recently, a flurry of studies in human cells demonstrated the existence of DSGs in newly synthesized DNA by employing EdU pulse-labelling experiments coupled to super-resolution microscopy [69] or by employing a modified DNA fiber assay coupled to S1-nuclease digestion [11,60,69,89,90,91]. Single-stranded DSGs within DNA fibers are sensitive to S1-nuclease activity and can thus be indirectly measured by the extent of DNA fragmentation [92]. The high frequency of DSGs detected in cells depleted for BRCA2 or other RAD51 mediator proteins (RAD51 paralogs) reinforced earlier observations that DSGs are repaired by HR in a RAD51-dependent manner [69,81,93]. DSG formation is induced by common DNA lesions, causing discontinuous DNA replication (e.g., unrepaired base damage caused by oxidative stress, UV-induced pyrimidine dimers, bulky DNA adducts induced by carcinogens, such as benzo-[a]-pyrene metabolites and other forms of DNA base damage) as well as DNA repair processes behind the fork (e.g., nicking of the sugar-phosphate backbone during base excision repair, nucleotide excision repair, and mismatch repair) [83]. Of note, DNA nicks or single-strand breaks (SSBs) ahead of replication forks cause the formation of one-ended DSBs due to a collision of replication forks with SSBs (also known as ‘replication run off’) [6,94,95]. Such one-ended DSBs essentially undergo HR- or BIR-mediated repair and require the restoration of a functional replication fork [6].

Mechanistically, DSG formation behind the replication fork requires priming activities of a dedicated primase, PRIMPOL, or a transient fork reversal transferring the DNA lesion and DSG behind the fork (classical models of UV repair) [83]. Moreover, uncoupling of replicative polymerases from a CMG helicase within the replisome can also give rise to DSGs behind the fork. The PRIMPOL-mediated stress tolerance pathway is activated during adaptation to chemotherapeutic treatments (e.g., in response to Cisplatin and other bulky DNA lesion-causing agents) and, at the same time, complementary to other stress responses such as TLS or fork reversal [89]. In particular, it was demonstrated that a single high dose of Cisplatin favors fork reversal in BRCA-deficient cells, whereas a low-dose Cisplatin treatment prior to a high dose resulted in adaptive stress responses favoring repriming and the ensuing generation of DSGs [89].

**Figure 3:**
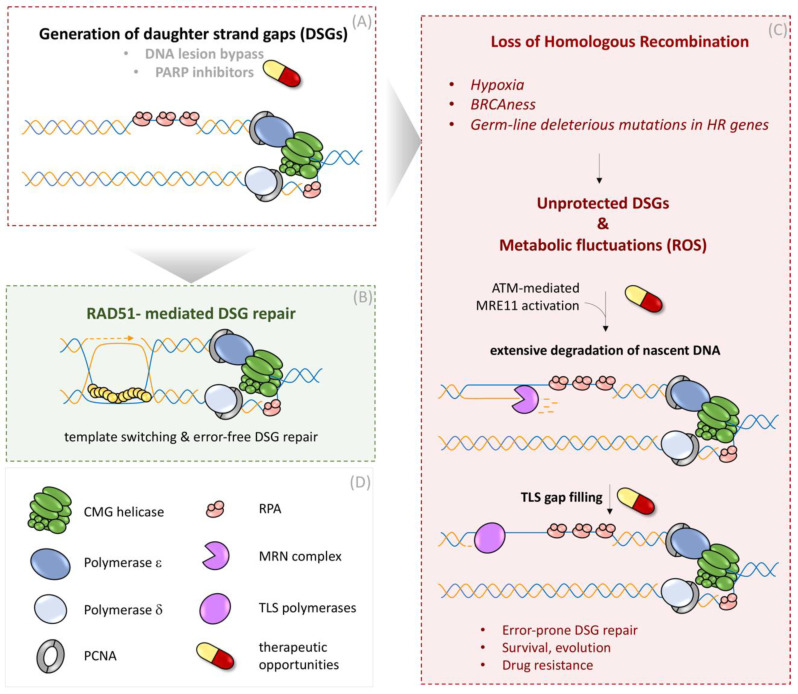
Pathophysiology of defective HR-mediated DSG repair. (**A**) The generation of daughter strand gaps (DSGs) can occur due to discontinuous replication, represented by re-priming events or caused by chemotherapeutic PARP inhibitors (for more details, see Figure 2). (**B**) In the presence of RAD51 and its mediators, DSGs undergo RAD51-dependent template switch repair (shown in Figure 2). (**C**) Permanent or transient loss of HR factors (e.g., BRAC2 or RAD51) by hypoxia, ‘BRCAness conditions’ (phenocopy of germ line *BRCA1* or *BRCA2* mutations), or hypomorphic cancer mutations in HR genes. In the absence of RAD51 or its mediators, DSGs remain unprotected. In combination with increased ROS levels (e.g., metabolic imbalances, hypoxia, or Hydroxyurea (HU) treatment) ATM activation can induce MRE11-dependent degradation of nascent DNA at unprotected DSGs. Translesion synthesis (TLS) polymerases fill up extended gaps in an error-prone manner, fueling mutation rates at such sites. Mechanistic findings can be therapeutically exploited in cancer cells that naturally proliferate with high replication stress as well as metabolic alterations. (**D**) Legend of symbols for respective DNA repair enzymes.

Complementary to the work with exogenous DSG inducers, our group studied the occurrence of DSGs during unperturbed DNA replication without any external interventions [69]. We demonstrated that stochastic bypass of DNA lesions and DSG occurrence is common during normal DNA replication and that DSGs are continuously surveyed, protected, and repaired by BRCA-RAD51 proteins behind the fork [69]. Furthermore, disruption of the BRCA-RAD51 pathway during oxygen starvation (hypoxia), which naturally repress HR proteins [96], switches the DNA repair synthesis in DSG from error-free, HR pathway to a rather error-prone, gap-filling mechanism by TLS polymerases [69] (Figure 3). Aligned with these findings, recent reports from several groups reported that DSGs are the prime source of genome instability in HR-deficient cells and that these structures underlie the chemosensitivity of BRCA1/2 mutant cells to TLS and PARP inhibitors [83]. By employing high-content and super-resolution imaging of RAD51 in the context of active DNA replication, we also established the occurrence of spontaneous RAD51 foci in unperturbed S-phase cells as a robust and specific marker for ongoing DSG repair [69]. This is consistent with recent work employing the ssDNA-binding RPA complex as a reporter for the DSG repair generated by polymerase-blocking lesions in yeast [97]. The comparison of different mammalian cell lines suggested that cancer cells show highly pronounced levels of spontaneously arising RAD51 foci compared to non-cancerous cells, highlighting an endogenous burden of replication stress in cancer cells featured by poor quality of template DNA, frequent lesion bypass, and increased DSG formation [69].

Curiously, while spontaneous RAD51 foci that mark DSG repair are formed in the mid/late S phase, a sizeable fraction of those foci is also present in G2 phase [69], raising the possibility that RAD51 filaments at specific genomic or chromatin contexts are preferably stabilized throughout S phase and might undergo resolution only after the bulk of DNA duplication is completed. Such a model, in which specific HR activities are synchronized with cell cycle stages, is supported by the finding that RAD54’s function in RAD51 removal from late HR intermediates is predominantly activated in the late S/G2 phase [98]. Indeed, a recent report from Vindigni and colleagues implicated two temporally distinct pathways of DSG filling in human cells, one exploiting RAD51 pathway in S phase and the other relying on the RAD18 in G2 phase [99]. More evidence for such an orchestrated repair of DSGs in synchrony with the cell cycle emerges from the observation that nucleases, which resolve late HR intermediates, are predominantly active in late G2/mitosis [79]. Collectively, these findings underline the importance of RAD51 stabilization on replication-born DSGs, which is crucial for protection and integrity of nascent DNA arising during perturbed or unperturbed DNA duplication.

#### 3.3.2. Expansion of Unprotected DSGs as a Source for Genome Instability

The pathophysiological relevance of defective or attenuated HR emerges at multiple levels, e.g., in patients with hypomorphic BRCA2 mutations who are prone to develop ovarian or breast cancers [16]. On a cellular level, similarly to what is observed after BRCA2 depletion [69], BRCA2 hypomorphic missense variants could fail to efficiently load RAD51 on ssDNA gaps. Pathophysiological consequences of inefficient protection of ssDNA substrates are also observed in cells exposed to hypoxic conditions marked by the loss of a variety of HR proteins including BRCA2, RAD51, and Rad51 paralogs [69]. As a result of perturbed RAD51 loading during hypoxic or BRCA2-hypomorphic conditions, replication-born DSGs remain unrepaired and accumulate in S-phase cells [69]. The generation of DSGs during hypoxia is not necessarily a result of an adapted replication stress response as shown for chemotherapeutic treatments [89], but rather stems from stochastic replication errors, which occur during every cell cycle [69].

Unprotected DSGs in HR-deficient settings represent vulnerable structures for nucleolytic attempts. Certain cellular conditions that inflict metabolic imbalance can hyper-activate nucleases and, thus, trigger pathophysiological degradation of nascent DNA, leading to extending the stretching of ssDNA within DSG. These conditions include but are not limited to metabolic fluctuations during hypoxic growth [69] or HU treatment [48,69], causing the increase of ROS levels, resulting in ROS-dependent ATM activation and down-stream stimulation of MRE11’s exo-nuclease activity [69], the major executor of DSG extension [88]. Consequently, the combination of unprotected DSGs during HR deficiency and the parallel nucleolytic activation by ROS emerge as a major source of nascent DNA instability [69]. Moreover, we found that extended DSGs can serve as substrates for TLS polymerases REV1-POLζ, Poli, Polk, and Poln, resulting in mutagenic gap-filling reactions [69] (Figure 3). The identification of unrepaired and extensively processed DSGs as an outcome of HR deficiency reveals thus far unappreciated mechanistic parallels between HR-mediated repair and the protection of nascent DNA, which are implicated as two distinct mechanisms in the context of stalled replication forks [66,100].

Of note, besides our experimental conditions during hypoxic growth, sources for increased cellular and nuclear ROS can be manifold and, for instance, can originate from the natural accrual of oncometabolites [101] or oncogene activation during cancer development [102]. Thus, we postulated that even mild ROS fluctuations during vulnerable states or transitions marked by inefficient DSG protection can fuel mutagenesis and genome instability by employing error-prone, gap-filling polymerases.

#### 3.3.3. Implications of DSG Repair for Genome Integrity Maintenance

Fanconi anemia proteins are involved in a series of functions during homology-directed repair including stabilization of RAD51 on ssDNA [103]. Besides increased genomic instability, one prominent phenotype in FA patients is bone marrow failure marked by decreased activity of hematopoietic stem cells and the resulting diminished blood cell counts [103,104]. Interestingly, hematopoietic stem cells are exposed to episodes of hypoxic growth within the anaerobe bone marrow microenvironment [105]. The absence of functional HR in FA patients in combination with hypoxia-induced increased ROS levels and high ATM-MRE11 activity might fuel genomic instability and dysfunction of hematopoietic stem cells in such patients. In such a scenario, extensive processing of DSGs could conspire to fuel RS and DNA damage accumulation that has been causally linked to an exacerbated p53/p21 response, thereby leading to hematopoietic stem cell elimination and bone marrow failure in FA patients [106].

Of note, other than cancer cells exposed to hypoxic periods during oncogenic transformation, stem cells do not suffer from depletion of HR proteins during hypoxic growth, e.g., during early embryonic development, which highlights the importance of protective HR functions and possible DSG repair, especially for embryonic stem cells [107]. Indeed, ESCs’ cells proliferate with high levels of replication stress, characterized by the accumulation of ssDNA replication gaps and massive levels of RAD51 loading on chromatin [107].

Given the heterogeneity of biological systems, we also considered the possibility that a subfraction of aberrantly extended DSGs behind the replication fork might escape gap-filling reactions by TLS polymerases, depending on chromatin context or secondary DNA structures. In such cases, DSGs might be transmitted over mitosis to the next G1 phase of the next cell cycle. Transmitted DSGs might resemble structurally inherited DNA lesions, which normally arise from DNA under-replicated loci in the previous cell cycle. These inherited DNA lesions sequester 53BP1 and other genome caretakers around under-replicated DNA lesions, resulting in the appearance of so-called 53BP1 nuclear bodies [108]. We speculated that extended DSGs might be part of such inherited DNA lesions, supported by the finding that BRCA2-deficient cells are marked by increased incidence of 53BP1 nuclear bodies [100]. This poses an important question of whether repair of inherited DSGs requires sophisticated mechanisms, the nature of which is currently not known. An important step in this direction is the recent work from Lee Zou’s laboratory, where the authors attributed PARP inhibitors’ effectiveness in eliminating BRCA1/2-deficient cells to the fact that PARP1 trapped on DSGs in the absence of HR is transmitted to daughter cells, where transmitted DNA lesions are converted to toxic DSBs [109].

## 4. Conclusions

In conclusion, the sheer variety of metabolic and genetic conditions in which the metabolism and repair of DSGs is altered or disturbed can be manyfold. Since replicative cells face such replication-associated DNA lesions every single cell cycle, the mounting attention towards DSGs, their formation, and their repair is justified and needs further investigations to fully understand their impact and involvement in various disease phenotypes and, at the same time, will open new windows of opportunity to exploit such DNA lesions for new or improved therapeutic interventions.

## Data Availability

Not applicable.
